# Toward a Nonspeech Test of Auditory Cognition: Semantic Context Effects in Environmental Sound Identification in Adults of Varying Age and Hearing Abilities

**DOI:** 10.1371/journal.pone.0167030

**Published:** 2016-11-28

**Authors:** Valeriy Shafiro, Stanley Sheft, Molly Norris, George Spanos, Katherine Radasevich, Paige Formsma, Brian Gygi

**Affiliations:** 1 Department of Communication Disorders and Sciences, Rush University Medical Center, Chicago, Illinois; 2 Veterans Affairs Northern California Health Care System, Martinez, California; University of Nevada Las Vegas, UNITED STATES

## Abstract

**Objective:**

Sounds in everyday environments tend to follow one another as events unfold over time. The tacit knowledge of contextual relationships among environmental sounds can influence their perception. We examined the effect of semantic context on the identification of sequences of environmental sounds by adults of varying age and hearing abilities, with an aim to develop a nonspeech test of auditory cognition.

**Method:**

The familiar environmental sound test (FEST) consisted of 25 individual sounds arranged into ten five-sound sequences: five contextually coherent and five incoherent. After hearing each sequence, listeners identified each sound and arranged them in the presentation order. FEST was administered to young normal-hearing, middle-to-older normal-hearing, and middle-to-older hearing-impaired adults (Experiment 1), and to postlingual cochlear-implant users and young normal-hearing adults tested through vocoder-simulated implants (Experiment 2).

**Results:**

FEST scores revealed a strong positive effect of semantic context in all listener groups, with young normal-hearing listeners outperforming other groups. FEST scores also correlated with other measures of cognitive ability, and for CI users, with the intelligibility of speech-in-noise.

**Conclusions:**

Being sensitive to semantic context effects, FEST can serve as a nonspeech test of auditory cognition for diverse listener populations to assess and potentially improve everyday listening skills.

## Introduction

When sensory information is degraded due to external signal distortions or peripheral sensory limitations, listeners tend to increasingly rely on contextual information to maximize the accuracy of perceptual decisions [1, 2]. The effective use of context in real-world listening tasks relies on cognitive abilities for efficient memory access and integration of semantic information across different object categories [3, 4]. In speech perception under adverse listening conditions, older and hearing-impaired adults often rely on semantic context to compensate for sensory limitations [5, 6]. Clinical tests have been developed and extensively used to evaluate the involvement of semantic context in speech perception [5–9], but are currently lacking for other meaningful sounds in listeners’ surroundings. Environmental sounds are an integral part of the everyday listening experience and can convey critical information for individual safety and well-being [10]. Previous research has demonstrated varied effects of semantic context in environmental sound perception in normal-hearing adults and children [11–15]. However, time-efficient tests for the assessment of nonspeech semantic context effects have not as of yet been developed. The purpose of the present study was twofold: a) to develop a brief test of context effects in environmental sound perception, suitable for both clinical settings and research endeavors, and b) using this test, evaluate the use of nonlinguistic semantic context in diverse listener populations.

### Environmental Sounds

In daily life, sounds rarely occur in isolation. The tacit knowledge of how they relate to one another informs the listener about what is happening in their environment, with significant implications for individual safety and quality of life [16–18]. Environmental sounds are produced by biological agents or inanimate objects and contain information about their sources. Normal-hearing listeners are able to accurately identify the sources of a large number of common environmental sounds, often in great detail [19,20]. For instance, they can identify the gender and posture of a walker [21,22], judge the fullness of a vessel as it is filled with water [23], the length of a rod after it was dropped on the floor [24], or accurately predict the timing of the bounces for different types of balls [25]. In addition to safety concerns associated with specific sounds (e.g., alarms, gun shots), environmental sounds also provide listeners with a sense of connection to the environment and aesthetic satisfaction [10].

As an ontologically broader class of ecologically relevant sounds, environmental sounds are different from speech in that they are nonlinguistic in nature. With the exception of alarms or other electronically synthesized sounds such as auditory icons specifically constructed to convey information, environmental sounds represent unintentional byproducts of distal events [16,17]. As such, they are also different from electronically produced psychoacoustic or laboratory test sounds designed to investigate perceptual capacity, rather than transmit information about the environment. They can be further distinguished from music which has an aesthetic intent and a generally more restricted range of sound sources. Successful perception of environmental sounds and auditory scenes requires integration of low-level sensory information with high-level cognitive representations from memory of distal objects and events [3]. Thus, as auditory stimuli, environmental sounds represent a useful contrast to speech, music and laboratory-generated acoustic stimuli in evaluation of the interaction between the sensory and cognitive aspects of auditory perception [26–30].

### Context Effects with Environmental Sounds

Previous research with normal-hearing young adults indicates that the identification of individual environmental sounds in an auditory scene can be affected by their contextual relationships. Similar effects have been previously found in the perception of objects in visual scenes [31, 32]. Over time, the causal dependencies and arbitrary but consistent probabilistic contingencies among sound-producing objects and events in an auditory scene can form stable semantic memory networks which affect the identification of specific sounds. Listeners tend to rely on contextual information to resolve perceptual ambiguity about specific environmental sounds in a sound sequence [11, 12, 33]. For instance, an ambiguous sound can be perceived as ‘a fuse burning’ when preceded by the sound of a match being struck and followed by the sound of an explosion, but it is perceived as ‘bacon frying’ when preceded and followed by other kitchen sounds [11].

Furthermore, when asked to categorize environmental sounds, adult listeners tended to group them based on semantic relationships that could either include abstract object properties or draw on meaningful activities the sounds represent in everyday life, e.g., ‘getting the groceries’ [34–36]. Semantic connections are formed among sounds that are likely to occur together, which results in the formation of a semantic memory network [33], also referred to as an auditory schema [3, 37]. Similar to identification of objects in visual scenes [31, 32], the identification of one or more of individual sounds within such schemas or networks activates other elements, increasing the likelihood of their identification [3, 11, 12]. On the other hand, semantic incongruence between a specific sound and the other sounds in an auditory scene can also result in an identification advantage [13]. For instance, the sound of rooster crowing in an auditory scene of a hospital emergency room is more detectable than the same sound heard in a barnyard ambience. This effect, however, is level-dependent, and tends to reverse with a low sound-to-scene energy ratio [16, 38].

Another recent investigation of how identification of individual sounds forming auditory scene-like sequences is affected by the contextual relationships among the sounds was undertaken by Risley and colleagues [14]. Young normal-hearing listeners were presented with 44 five-sound sequences. Half the sequences were formed from contextually coherent sounds (i.e., semantically related sounds likely to be heard at the same place and time) and half were not. The same individual sounds were used in both coherent and incoherent sequences to ensure that differences in identification performance were not driven by differences among specific sounds. Consistent with earlier findings, results indicated that sound identification was significantly better when the sounds were embedded in contextually coherent rather than incoherent sequences.

### Aging, Cognitive Function and Hearing Loss

Aging has been shown to be associated with a decline in cognitive abilities, including working memory and attention [39–41]. A decline in hearing abilities that frequently accompanies aging can influence peripheral encoding of auditory information. A number of recent studies have reported an association between hearing and cognitive abilities [42–46]. Although the underlying mechanisms are not well understood [2, 47], it is likely that the fidelity of sensory information can influence the use of the semantic context in auditory scenes. However, there is limited knowledge about the extent to which older adults, either with or without a hearing loss, utilize nonlinguistic semantic auditory information for sounds other than speech.

Evidence from speech research that has evaluated the effect of semantic context indicates that in adverse listening environments older and hearing-impaired adults tend to rely on context to a greater extent than younger or normal-hearing listeners[7, 48, 49]. This compensatory strategy helps to maintain adequate speech intelligibility despite a decrease in the quality and quantity of sensory information. However, when the semantic context in sentences is limited, intelligibility declines and substantial effects of listener age and hearing loss often emerge. It is likely that similar compensatory processes may be an aspect of environmental sound perception in older normal-hearing and hearing-impaired adults.

### Cochlear Implants

Cochlear implants (CIs) have provided a highly effective intervention for hearing-impaired individuals who do not benefit from other types of sensory aids. However, a number of perceptually salient acoustic features are removed (e.g., temporal fine structure) or severely degraded (e.g., spectral resolution) by the signal processing of the implant [50]. Distortion due to signal processing can compound other user-specific impediments to environmental sound perception associated with age, language development, history of deafness and cognitive status. As a group, even experienced postlingual adult CI users with good speech recognition demonstrate difficulty in the identification of many common environmental sounds that tend to lack a distinct energy envelope, or are typically low-level (e.g. ‘zipper’, ‘blowing nose’, ‘thunder’) [51–55]. Furthermore, due to prolonged deafness often preceding implantation, a large numbers of CI users cannot determine the sources of many environmental sounds associated with daily activities (e.g., ‘car horn’, ‘water running’). It appears that even when sensory stimulation provided by the implant is sufficient for accurate environmental sound identification, many CI users may misinterpret it, attributing potentially meaningful sounds to generic background noise. However, environmental sound recognition by CI users can quickly improve following training and instruction, presumably when the sensory input is remapped to the correct source properties stored in listeners’ memory [55]. This fast learning may be akin to normal-hearing listeners quickly learning to recognize speech in a noise-vocoded signal after hearing only a few words [56]. The general lack of awareness about sources of specific sounds in the environment, confounded with a potentially compromised ability to effectively manipulate auditory objects in working memory [57], may further interfere with the ability of implant users to utilize the semantic information in auditory scenes. However, without appropriate assessment tools, these potential deficits are difficult to address.

### Present Study

The present study was designed, first, to develop a clinical test of auditory cognition based on nonlinguistic environmental sound stimuli—a test suitable for use in both clinical and research settings with diverse patient populations. The second goal was, using this test, to extend previous investigation of context effects in environmental sound perception by examining the role of aging, hearing impairment and cochlear implants. To that end, in Experiment 1, a short test of environmental sound sequences was constructed from the stimuli used in previous environmental sound studies [14, 19, 20, 30, 58]. The sensitivity to contextual relationships among the individual sounds of the revised shorter test was confirmed with young normal-hearing (YNH) adults who served as a control group. To investigate the effect of aging and hearing loss, in Experiment 1 the test was also administered to middle-to-older aged listeners with either normal hearing or a mild-to-moderate sensorineural hearing loss. Experiment 2 investigated the effect of CI processing on the ability to utilize contextual information conveyed by environmental sounds by using two additional groups: YNH adults tested with a vocoder CI simulation and experienced CI users. It was expected that in both experiments all listener groups would demonstrate significantly better performance with contextually coherent than incoherent sound sequences.

## Experiment 1

This experiment investigated the influence of aging and hearing loss on the involvement of semantic context in environmental sound perception. Based on findings from earlier work with YNH adults [11, 12, 14, 59] and results from speech perception research [5, 49, 60,61], young and older listeners were expected to demonstrate greater accuracy when identifying sounds in contextually coherent scenes than with incoherent scenes. Further, it was hypothesized that while both older listeners and YNH adults would benefit from contextually coherent scenes, the overall performance accuracy for contextually incoherent sequences would be higher for YNH adults.

### Methods

#### Ethics Statement

All methods were approved by the Institutional Review Board of the Rush University Medical Center, and all participants provided written informed consent.

#### Stimuli and Procedure

Upon completing a background audiometric assessment, subjects completed the environmental sound test. Participants in the the middle-to-older aged normal-hearing (MON) and the middle-to-older aged hearing-impaired (MOI) groups were also assessed for speech perception in noise, cognitive status and working memory ability. Environmental sounds were presented diotically at 75 dB SPL. The levels used in speech testing are described below. All auditory testing was conducted in a double-walled soundproof booth using Etymotic ER-3A insert earphones. After completing the cognitive tests, subjects received audiometric evaluation and speech testing, and concluded with the environmental sound tests.

Environmental sound perception was tested with the following. Familiar Environmental Sound Test—Identification (FEST-I) comprised sounds selected from a previously developed large-item test of environmental sound perception [19, 20, 30, 58]. Twenty five familiar environmental sounds were used in the current short-form testing (Table 1). Based on the results of previous studies, these 25 sounds were found to be highly identifiable by YNH listeners. The test sounds were normalized in root-mean-square (RMS) energy, after being corrected for silent pauses. They were presented to subjects one at a time in random order, using a desktop computer with a graphical user interface to collect listener responses. After hearing each sound, subjects selected from one of the 25 sound names, displayed alphabetically on the screen, that best described the sound just heard, at which point the next sound was played. To familiarize listeners with response options, prior to testing listeners were given a picture of the screen with all the sound names as they appeared during testing, and were asked to read all labels aloud. During the test, subjects were encouraged to guess when they were not sure about sound identity. On average, FEST-I administration took about 5 minutes.

**Table 1:** Composition off the Familiar Environmental Sound Test for individual sounds (FEST-I) and sound sequences (FEST-S).

Sound Name	Duration (sec)	Sequence Name (C)	Position (C)	Sequence Name (I)	Position (I)
alarm ringing	4.74	Waking up	3	INC05	3
barking	1.23	House visitor	2	INC03	3
birds chirping	1.18	Waking up	5	INC02	4
fog horn	3.97	Ocean side	2	INC02	2
tires screeching	1.37	Car accident	2	INC01	2
busy signal	2.55	Phone call	5	INC05	5
crashing	4.85	Car accident	4	INC03	5
dialing	5.26	Phone call	4	INC04	1
dial tone	2.09	Phone call	3	INC02	3
doorbell	1.93	House visitor	1	INC04	4
door closing	2.06	House visitor	4	INC02	1
driving	2.17	Car accident	1	INC02	5
honking	0.92	Car accident	3	INC04	2
dog panting	2.18	House visitor	5	INC05	2
phone ringing	2.93	Phone call	1	INC03	2
pickup receiver	0.56	Phone call	2	INC01	4
rooster	1.64	Waking up	2	INC04	3
seagulls	1.98	Ocean side	1	INC05	4
police siren	3.88	Car accident	5	INC05	1
snoring	3.72	Waking up	1	INC03	4
splash	1.96	Ocean side	5	INC03	1
trotting	6.58	House visitor	3	INC01	5
footsteps	5.04	Ocean side	4	INC04	5
waves crashing	2.55	Ocean side	3	INC01	3
yawning	2.38	Waking up	4	INC01	1

The 25 sounds of FEST-I and their durations along, with each sound’s position in the coherent (C) and incoherent (I) sequences of FEST-S. All environmental sound stimuli are available online in wav format and can be downloaded from https://zenodo.org/record/59186. A Matlab software package used for presenting FEST stimuli can be downloaded from https://zenodo.org/record/59187

Familiar Environmental Sound Test—Sequences (FEST-S)—the 25 individual sounds used in FEST-I were arranged into two separate sets of five sequences, with each sequence composed of five individual sounds (Table 1). The two sets of sequences differed in their contextual coherence rating obtained in previous work by Risley and colleagues [14]. For one set of sequences, the individual sounds in each of the five sequences were highly contextually coherent, representing an auditory scene that was judged as being likely to be heard in the world. In the other set, the sounds in each sequence were contextually unrelated, and judged as not likely to be heard together. However, both contextually coherent and incoherent sets of sequences were constructed from the same 25 individual sounds, but differently arranged. In both coherent and incoherent sequences, individual sounds were separated by silent intervals of 250 ms. Overall durations of the FEST-S sequences varied between 12 and 17 seconds.

Individual FEST-S sequences were presented in random order. After hearing all five sounds in each sequence, subjects first rated its contextual coherence, i.e. how likely the sounds in the sequence were to be heard at the same place and time. Subject responses were entered using a slider with minimum (0.0) and maximum (1.0) scale values representing “extremely unlikely” and “extremely likely,” respectively. Next, subjects selected the names of the sounds from the 25 names on the screen and arranged them in the order they were presented in the sequence. Subjects were free to begin the sound identification task from any position in the sequence between the first and last sound. Sequence presentation was subject paced. Two practice trials using one coherent and one incoherent sound sequence were completed as many times as necessary to ensure understanding of the task (typically once or twice). The environmental sounds of the practice trials were not used in the scored FEST-S testing. Prior to testing, each subject read the list of 25 environmental sounds out loud to ensure familiarity with all sound name options and their location on the display. On average, FEST-S administration took about12-15 minutes. The Matlab software package used for presenting FEST stimuli can be downloaded from https://zenodo.org/record/59187

Environmental sound context effects assessed by FEST-S involve manipulation of individual sounds in working memory. Although FEST-S is administered in the auditory modality and thus involves auditory working memory, it could also be expected that manipulations across semantic categories may involve general modality-independent aspects of working memory. To test this possibility, MON and MOI subjects were given two working memory tests, one delivered through the auditory and the other through the visual modality.

Letter-Number Sequencing (LNS), presented to subjects aurally, is a subscale of the WAIS test [62] in which subjects are read random strings of letters and digits. Their task is to report back the letters and digits they just heard as an organized sequence, first the numbers in ascending order followed by the letters in alphabetical order. If the subject responds correctly, the number of letters and digits gradually increases until a maximum number of letters and digits is reached, which represents the test score.

The Reading Span (RS) test assesses parallel operations of both memory storage and semantic processing abilities [63, 64]. It involves visual presentation of sentences, word by word, on a computer screen. Upon presentation of each sentence, the subject decides whether the sentence is semantically reasonable or not (e.g., “The horse sang loudly” vs. “The girl played on the beach”). After a number of sentences are presented, the subject is asked to repeat either the first or the last word that occurred in each of the sentences presented (without knowing in advance which word will be required). The number of sentences presented in one sequence before recall is gradually increased from three to six, with each condition (defined by number of sentences) repeated three times. The number of words correctly recalled across all sentences is the reading span score.

The Montreal Cognitive Assessment (MoCA) [65] is a brief neuropschological test used to provide a broad assessment of the cognitive status of several sub domains, including working memory, attention and executive function. It includes linguistic (naming), visual-motor (trail making and visuoconstruction skills) and aural tasks (word recall, forward and backward digit span) which are scored to a total maximum of 30 points. Since MoCA was originally designed as a cognitive screening instrument, it was not administered to the YNH study participants who, as students in a post-baccalaureate graduate program, tend to produce ceiling performance precluding further analysis.

Two tests were used to evaluate speech perception in noise. The first assessed speech perception in terms of the intelligibility of sentences from the Quick Speech-in-Noise Test (QuickSIN) in the presence of a four-talker speech-babble masker [66]. Each QuickSIN list contains six sentences with signal-to-noise ratio (SNR) decreasing in 5-dB steps from 25 to 0 dB across sentences. Based on the number of key words correctly repeated, results were converted to the metric SNR Loss, the estimated SNR needed for 50% correct relative to the performance of normal-hearing young adults. This metric thus represents a normalized speech reception threshold. Two scored lists were used along with a single practice list. QuickSIN testing was conducted with diotic presentation. Following the clinical protocol recommended by the test developer, diotic presentation level was 70 dB HL for the target speech.

The second measure was based on the Speech in Noise Test—Revised (SPIN-R) [8], used to assess the effect of semantic context in final-word identification in background noise. Testing used a list of 50 SPIN-R sentences spoken by a male talker in the presence of Gaussian noise lowpass filtered at 8 kHz. The signal-to-noise ratio (SNR) was 0 dB, with the speech at 75 dB SPL. In half of the sentences, the final word was highly predictable from the semantic context of the sentence, while in the other half, the word was not easily predictable. Using a graphical computer interface, after listening to each sentence, subjects selected one of the 50 words shown on the screen, as in previous studies [55, 56, 67].

#### Subjects

**Fig 1:**
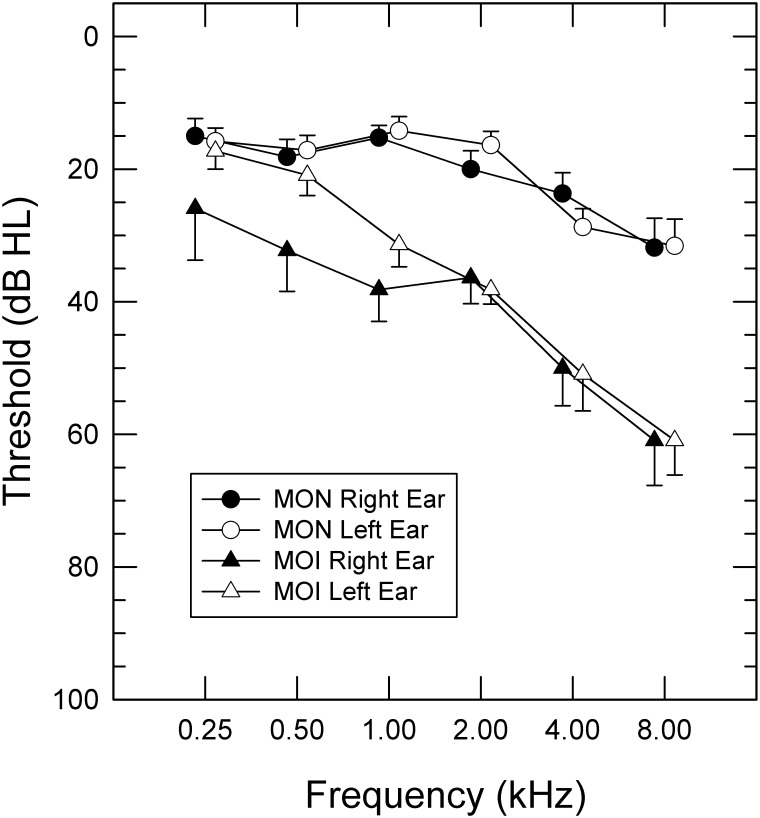
Audiometric thresholds. Audiometric thresholds for each ear for middle-to-older-normal-hearing (MON) and middle-to-older hearing-impaired (MOI) participants. The error bars represent 1 standard error shown on one side of each curve for better visibility.

Three groups of listeners participated in Experiment 1. The first group consisted of 15 young adults (five males; age range: 21–28 yrs; mean: 24.4 yrs) who had normal audiometric thresholds (≤15 dB HL re: ANSI 2004) for the octave frequencies between 0.25–4.0 kHz. The second and third groups consisted of middle-to-older aged listeners, with the groups distinguished by the pure-tone average (PTA) of audiometric thresholds between 0.25–4.0 kHz for their better hearing ear. There were 19 participants in the older MON group and 11 participants in the older MOI group. The subjects in the MON group (5 males; age range: 54–78 yrs; mean: 63.1 yrs) had an average PTA of 16.6 dB HL (*SD* = 6.9 dB). Subjects in the MOI group (5 males; age range: 53–75 yrs; mean: 66 yrs) had an average PTA of 33.4 dB HL (*SD* = 6.7 dB), with the hearing loss confirmed as sensorineural by bone-conduction thresholds and tympanometry. For all but two of the MOI participants, hearing loss was symmetric with less than a 7-dB between-ear difference in PTA. For the remaining two participants, the between-ear PTA difference was 15 and 43.7 dB. Group audiograms for the MON and MOI listeners are shown in Fig 1. Despite labeling of the MON group as older normal-hearing listeners, all but five of the participants exhibited at least a mild hearing loss at 8 kHz in at least one ear. All but one of the study participants spoke English as their native language. The one participant in the MOI group for whom English was not a native language was highly fluent in English and his environmental sound test scores were above the median of his group. Therefore his data was analyzed with the other participants.

Unlike FEST-S that was administered to all participants, FEST-I was administered to 17 subjects from the MON and MOI groups. It was added after the initial 13 subjects in MON and MOI groups were already tested as an extra precautionary measure to confirm high identification of the individual sounds in older listeners. Although performance on coherent and incoherent FEST-S sequences is based on the same set of individual sounds, which prevents individual sound identification from confounding performance differences between coherent and incoherent sequences, FEST-I scores were obtained to verify high identification accuracy for individual sounds in the two older groups. Thus, FEST-I was administered only to 11 and six of the MON and MOI participants, respectively. It was not administered to YNH group since previous studies consistently indicated high (above 90% accuracy) with these test sounds.

### Results and Discussion

Environmental sound results were analyzed to determine the role of semantic context in the perception of individual environmental sounds and to examine the relationships of environmental sounds with working memory and speech perception abilities. Initially, the coherence ratings for all sound sequences were examined in all groups to confirm the classification of sequences into the two different categories. Next, individual sound identification scores obtained in FEST-I were examined in a sample of listeners in the MON and MOI groups to verify their familiarity with the test stimuli. In turn, results from environmental sound sequence test, FEST-S, were evaluated using three outcome metrics: labels correct (LC), order correct (OC) and sequence correct (SC). These three metrics applied to the same listener responses, but differed in how stringently the responses were evaluated. For the LC metric, a response was counted as correct if the label chosen corresponded to any of the five sounds in the corresponding sequence. For the OC metric, a response was counted as correct only if a correct response label was placed in the correct position for the corresponding sound in the sequence. For the SC metric, a response was counted as correct only if all five sounds in a sequence were correctly labeled and each label was placed in the correct order of sound presentation. Thus, for the LC and OC metrics, there were 25 scored responses per condition (five sounds times five sequences), while for the SC metric, a single binary score was derived from the response to each trial. Finally, correlation analysis was performed to examine potential relationships among recognition of environmental sound sequences, speech-in-noise intelligibility, and working memory ability. In analyses, percent correct scores from the FEST-I, FEST-S, and SPIN-R tests were submitted to an arcsine transform before data analysis.

#### Coherence Ratings

All three groups rated the sounds of the semantically coherent sequences as being more likely to be heard in the same place or time than those in the incoherent sequences (Table 2). The mean rating of coherent sequences by YNH subjects was somewhat higher than the mean MON and MOI scores (0.92 vs. 0.78 and 0.74, respectively). In contrast, the mean rating of incoherent sequences by YNH listeners was lower than those of MON and MOI subjects (0.24 vs. 0.38 and 0.42, respectively). Although a repeated-measures analysis of variance (ANOVA) with three groups as a between subject factor and two levels of coherence as a within subject factor failed to reveal a significant effect of group [F(2, 42) = .002, p > .99, η_p_^2^ = 0], it indicated a small but significant interaction between group and coherence [F(2,42] = 4.8, *p <* .02, η_p_^2^ = 0.19]. Post-hoc multiple comparisons with Bonferrorni corrections failed to reveal any significance differences between specific group pairs. The ANOVA also confirmed a significant effect of coherence [F(1,42] = 86.8, *p* < .001, η_p_^2^ = .674]. Overall, these results suggest that despite some group variation in coherence judgments, suggested by the interaction, the three groups were comparable in their ability to distinguish between semantically coherent and incoherent sequences.

**Table 2:** Rating of contextually coherent and incoherent sound sequences.

	Experiment 1			Experiment 2	
	YNH	MON	MOI	CIV	CI
**Coherent Sequences**	0.92 (0.03)	0.78 (0.05)	0.74 (0.06)	0.69 (0.04)	0.79 (0.06)
**Incoherent Sequences**	0.24 (0.06)	0.38 (0.05)	0.42 (0.08)	0.26 (0.03)	0.33 (0.07)

Average rating of sound sequences by listeners groups in both experiments: young normal-hearing (YNH), middle-to-older normal-hearing (MON), middle-to older hearing-impaired (MOI) listeners, YNH subjects listening through vocoder-simulated implants (CIV) or cochlear-implants users (CI). Rating values increase with perceived coherence of the FEST sequences. Standard errors are shown in parentheses below the average rating for each condition.

#### Individual Sound Identification: FEST-I

Analysis of individual sound identification responses was conducted on a subset of 17 MON and MOI listeners indicated that listeners in both the MON and MOI groups were able to identify the 25 individual sounds in the test with high accuracy. The overall identification rate was 88.5% correct (*SD* = 9.8). Although mean MON accuracy was somewhat higher than that of the MOI subjects (91.3 vs. 83.3%, respectively), this difference was not significant in an independent-samples t test (*t* (15) = 1.58, *p* = .14).

#### Context Effects: FEST-S

**Fig 2:**
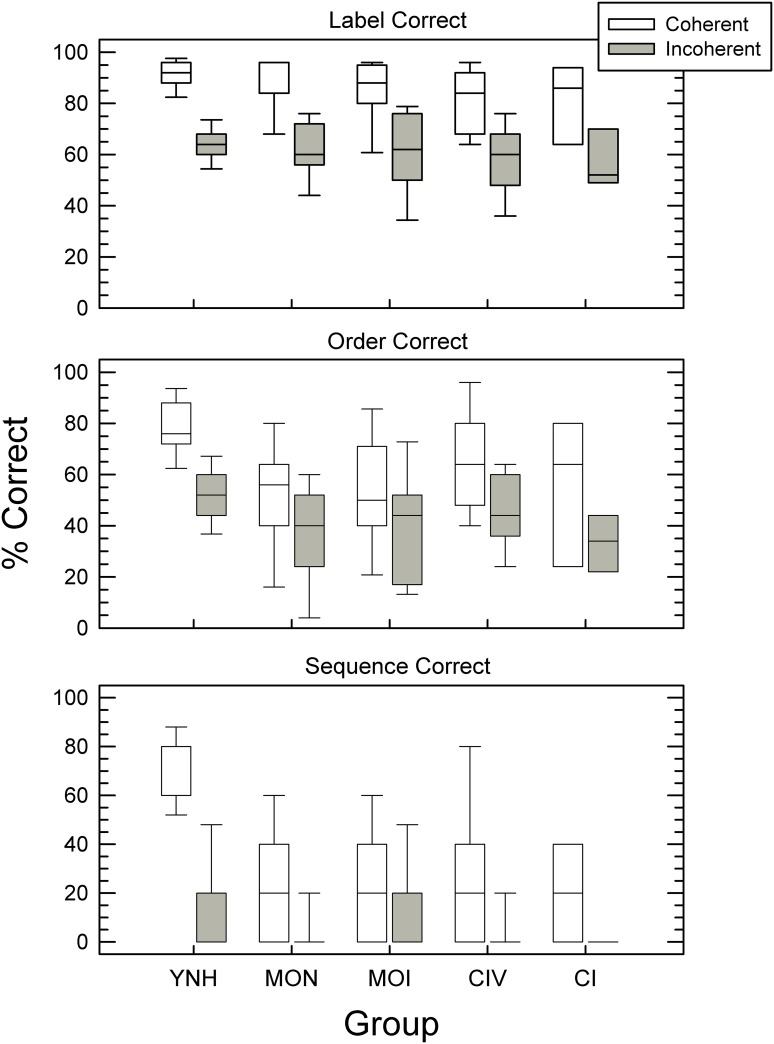
Performance accuracy on Familiar Environmental Sound Test—Sequences (FEST-S). As box plots, performance accuracy of each group for contextually coherent (open boxes) and incoherent sequences (gray boxes) for each of the three scoring metrics: Labels Correct (top), Order Correct (middle), and Sequence Correct (bottom). The line through each box is the median threshold; the upper and lower box edges indicate the 25th and 75th percentiles with error bars showing the 10th and 90th percentiles. Please note that with more stringent scoring metric of Sequence Correct, many listeners across groups did not respond correctly to any of the incoherent sequences, skewing the group distribution of scores. Consequently, the line displayed at 0% correct may represent the group median as well as the 25th and 75th percentiles of the performance distribution.

Analysis of context effects revealed that all three groups were able to benefit from contextual relationships among sounds in semantically coherent environmental sound sequences (Fig 2). For each of the three response metrics (LC, OC, SC), performance was higher for the semantically coherent sequences. To further evaluate these effects and assess their statistical significance, three separate repeated-measures ANOVAs were conducted—one for each response metric with context as a within-subject variable and group as a between-subject variable. A significant main effect of context was found for all three response metrics [(LC: *F*(1,42) = 241.38, *p* < .001, η_p_^2^ = .85; OC: *F*(1,42) = 84.62, *p* < .001, η_p_^2^ = .67; SC: *F*(1,42) = 65.24, *p* < .001, η_p_^2^ = .61), but the effect of group was significant only for the two more stringent metrics (OC: *F*(2,42) = 8.01, *p* = .001, η_p_^2^ = .28; SC: *F*(2,42) = 16.86, *p* < .001, η_p_^2^ = .45). Effect sizes of context were also greater than those of group. Post-hoc analyses with Bonferroni correction for multiple comparisons indicated that significant group differences adjusted to *p* < .016 (0.05/3) were between either YNH and MON or YNH and MOI groups, but not between the MON and MOI groups. Consistent with these significant effects of group, for the OC and SC metrics only, there was a significant interaction between group and context (OC: *F* (2, 42) = 3.34, *p* = .045, η_p_^2^ = .14; SC: *F* (2, 42) = 8.84, *p* = .001, η_p_^2^ = .30), indicating variation in the magnitude of the context effect across groups for these response metrics.

FEST-S scores averaged across all 10 FEST sequences for each group, without division by contextual coherence, indicated differences in performance between the YNH controls and both the MON and MOI groups. However, these differences emerged only with the more stringent metrics: OC and SC. With the most lenient scoring metric, LC, all three groups performed similarly on FEST-S overall: 77.6% (*SD* = 4.4) for YNH, 72.9% (*SD* = 11.8) for MON and 73.1% (*SD* = 13.9) for MOI. In contrast, for OC, the overall score of YNH listeners was 65.2% (*SD* = 8.2), while MON and MOI listeners’ overall scores were each 44.4% for OC (*SD* = 18.2 and *SD* = 20.3, respectively). For SC, YNH listeners’ overall scores were 43.3% (*SD* = 11.1), while MON scored 13.7% (*SD* = 13.4) and MOI scored 13.6% (*SD* = 13.6). These differences in results across the scoring metrics indicate an advantage for YNH over MON and MOI adults on aspects of the task that place greater demands on working memory.

The overall magnitude of the context effect was greater for YNH than MON and MOI subjects, whose performance, in turn, was remarkably similar. Furthermore, with the more stringent response metrics of OC and SC, the magnitude of context effects was smaller for MON and MOI subjects, compared to that with LC scoring. In contrast, for YNH listeners, the magnitude of the context effects nearly doubled with the SC metric compared to the LC or OC metrics (Fig 2), suggesting greater ability of YNH listeners to utilize contextual relationships among environmental sounds in auditory scene-like sequences. These differences in results among the scoring metrics indicate variation in the extent to which semantic context facilitates performance by different groups. Although all groups were able to utilize context information from surrounding sounds, YNH adults were able to do so more effectively.

#### Relationship of FEST-S Performance with Cognitive Status and Speech Perception

Overall, MON and MOI listeners demonstrated highly comparable results on the tests of cognitive abilities and speech perception in noise. Independent t-tests between the two groups revealed no significant differences on any of the tests. Both groups had very similar average scores on MoCA [MON: 26.5 (*SD* = 2.4), MOI: 25.7 (*SD* = 3.3), t(28) = .71, p = .48], and LNS [MON: 9.0 (*SD* = 2.4), MOI: 9.0 (*SD* = 3.1), t(28) = .05, p = .96]. Individual scores on MoCA of both MON and MOI participants were highly variable in the range of 21–30 and 20–30, respectively. Although several participants with such low MoCA scores would be considered at risk for dementia, they were kept in the present analysis because of their higher scores on other working memory tests, which increased the overall performance range of the sample. This allowed MoCA scores to be used descriptively along with other cognitive tests to examine factors that influence environmental sound performance. On the RS test, the groups were also not significantly different [t(28) = -1.39, p = .18]. However, the MON listeners on average performed somewhat lower [mean: 40.4 (*SD* = 8.9)] than the MOI participants [mean: 47.3 (*SD* = 18.8)], who also had greater variance. The larger standard deviation of the MOI group on the RS test primarily reflects the good performance of two participants whose scores were roughly 80. Similarly, the groups were comparable in terms of their ability to perceive speech in the presence of a multi-talker babble. There were no differences in their QuickSIN performance [t(28) = -.5, p = .62]. For the MON group, the mean SNR Loss was 2.7 dB (*SD* = 2.0), with a mean for the MOI group of 3.05 dB (*SD* = 1.7). No differences between the groups were also found for either low or high predictability SPIN-R sentences [t(28) = 1.6, p = .12] and t(28) = 1.7, p = .11, respectively]. For the MON group, the mean accuracy for low predictability sentences was 85% (*SD* = 8), while for high predictability sentences it was 97% (*SD* = 4). For the MOI group, the mean accuracy for low predictability sentences was 78% (*SD* = 13), while for high predictability sentences it was 93% (*SD* = 7). The lack of significant differences between MON and MOI groups on both QuickSIN and SPIN-R tests indicates that MOI listeners were able to maintain age-appropriate speech-in-noise perception despite their hearing loss.

Evaluation of the association between environmental sound sequence perception and tests of cognitive status and speech-in-noise abilities was conducted with multiple linear regression models that controlled for age and hearing sensitivity. Separate models were run for results obtained with coherent and incoherent sequences of the FEST-S protocol. Only one of the three FEST-S scoring metrics was used in the analysis since the three metrics are based on the same underlying data and are not independent. The OC metric was chosen because of the broader range in performance it provides compared to either the more or less stringent measures. The analysis was conducted with the MOI and MON groups combined due to lack of significant differences in their FEST-S performance.

For coherent sequences, the regression model was statistically significant [*F* (8, 29) = 3.99, *p* = .005], and as estimated by an adjusted R^2^ of .452, accounted for slightly over 45% of the variance in sequence recognition (Table 3). The regression model was also statistically significant [*F* (8, 29) = 4.76, *p* = .002] for incoherent sequences (Table 4), in this case with an adjusted R^2^ of .509. The control covariates of age and PTA did not significantly contribute to the prediction of either model. Among the speech and cognitive variables, overall cognitive status, as assessed by MoCA, was the only significant covariate in both models. For incoherent-sequence performance, a significant contribution was also obtained from LNS scores. Similar to the contrast between coherent and incoherent sequences in FEST-S, SPIN-R evaluates the effect of context on task performance. The lack of significant contribution from both SPIN-R metrics to either model prediction could in part be attributed to the overall high performance on both high and low predictability versions of the SPIN-R test, resulting in a compressed range between the two scores.

**Table 3:** Relation of coherent-sequence performance to age and auditory and cognitive abilities for older listeners.

Independent Variable	Estimate	*SE*	95% CI (lower/upper)	*β*	*p* value	Squared Bivariate Correlation	Squared Semi-Partial Correlation
**Age**	.000	.007	-.013/.014	.014	.950	.057	>.001
**PTA**	.005	.004	-.003/.014	.256	.195	.024	.034
**QuickSIN**	.014	.023	-.033/.062	.123	.535	.191	.008
**SPIN High Context**	-.105	.319	-.768/.558	-.074	.745	.079	.002
**SPIN Low Context**	.533	.298	-.087/1.153	.376	.088	.124	.060
**MoCA**	.050	.018	.013/.087	.625	.010	.364	.150
**LNS**	.020	.016	-.013/.053	.241	.215	.242	.031
**RS**	.000	.003	-.007/.007	.075	.941	.191	>.001

Relation of coherent-sequence performance for identifying sounds in the correct order to age, auditory and cognitive abilities for older listeners with and without hearing loss. Table entries are the estimated coefficient, standard error (*SE*), the lower and upper 95% confidence interval (CI) for the estimate, standardized coefficient (β), *p* value, squared bivariate correlation, and squared semi-partial correlation of a linear regression model predicting sequence performance. For the model, *F* (8, 29) = 3.99, *p* = .005, and R^2^ and adjusted R^2^ were .603 and.452, respectively.

**Table 4:** Relation of incoherent-sequence performance to age and auditory and cognitive abilities for older listeners.

Independent Variable	Estimate	*SE*	95% CI (lower/upper)	*β*	*p* value	Squared Bivariate Correlation	Squared Semi-Partial Correlation
**Age**	.001	.006	-.012/.015	.047	.817	.028	>.001
**PTA**	.002	.004	-.006/.010	.087	.636	.008	.004
**QuickSIN**	.014	.022	-.031/.059	.116	.536	.218	.007
**SPIN High Context**	-.319	.303	-.949/.312	-.224	.305	.048	.019
**SPIN Low Context**	.325	.284	-.265/.916	.228	.264	.116	.022
**MoCA**	.045	.017	.010/.080	.559	.015	.350	.120
**LNS**	.039	.015	.008/.070	.469	.016	.367	.117
**RS**	.002	.003	-.005/.008	.095	.628	.180	.004

Relation of incoherent-sequence order-correct performance to age, auditory and cognitive abilities for older listeners with and without hearing loss. Table entries are the estimated coefficient, standard error (*SE*), the lower and upper 95% confidence interval (CI) for the estimate, standardized coefficient (β), *p* value, squared bivariate correlation, and squared semi-partial correlation of a linear regression model predicting sequence performance. For the model, *F* (8, 29) = 4.76, *p* = .002, and R^2^ and adjusted R^2^ were .645 and.509, respectively.

As exploratory analyses, the linear regression models included covariates that assessed either similar abilities (e.g., SPIN Low and QuickSIN) or interrelated processing (e.g., involvement of working memory in speech-in-noise performance). Consequently, colinearity among model covariates was anticipated. This colinearity is illustrated for each independent variable by comparison of the squared bivariate correlation and squared semi-partial correlation. In both models, the semi-partial correlations are much lower (see Tables 3 and 4), indicating that the unique contribution of the covariate to the model prediction is much less than the variance accounted for by that variable in isolation as represented by the squared bivariate correlation. To further explore colinearity among model covariates, commonality analysis [68] was used to partition the multiple regression effects described in Tables 3 and 4 in terms of unique and shared variance. For both models, the sum of the unique contributions from the eight independent variables accounted for nearly half of the regression effect (coherent sequence model: 47.2%; incoherent-sequence model 45.5%), with the remaining variance components shared across two or more variables.

Tables 5 and 6 list the unique and shared variance components that contributed at least 6% to the variance explained in the regression model for coherent and incoherent sequences, respectively. The largest variance component in both models was the unique contribution of MoCA, with a roughly comparable unique contribution from LNS in the incoherent-sequence model. Apart from the unique contribution from SPIN Low in the coherent-sequence model (Table 5), the remaining variance components contributing at least 6% to the regression effects included multiple covariates, mixing the speech and cognitive metrics.

**Table 5:** Unique and shared variance components in the linear regression model for coherent-sequence performance.

Component	Variance explained (%)
**Unique: MoCA**	24.8
**Unique: SPIN Low**	10.0
**Shared: SPIN Low, LNS**	8.5
**Shared: Age, MoCA**	7.5
**Shared: QuickSIN, SPIN Low, MoCA, LNS**	7.2
**Shared: Age, QuickSIN, MoCA**	6.3

Unique and shared variance components contributing at least 6% to variance explained in the linear regression model of Table 3 for coherent sequences.

**Table 6:** Unique and shared variance components in the linear regression model for incoherent-sequence performance.

Component	Variance explained (%)
**Unique: MoCA**	18.6
**Unique: LNS**	18.1
**Shared: SPIN Low, LNS**	10.2
**Shared: QuickSIN, MoCA, LNS**	7.9
**Shared: QuickSIN, SPIN Low, MoCA, LNS**	7.1
**Shared: Age, MoCA**	7.1
**Shared: MoCA, LNS**	6.2

Unique and shared variance components contributing at least 6% to variance explained in the linear regression model of Table 4 for incoherent sequences.

Overall, the regression analyses indicated that MoCA, the summary measure of cognitive status, was significantly associated with FEST-S performance with both coherent and incoherent sequences. Significant association of auditory working memory as assessed by LNS was obtained only for the incoherent sequences of the FEST-S protocol. Effect of sequence type on the relationship to working memory is consistent with an absence of sequence context leading to greater memory demands. Though both environmental sound sequence recognition and speech-in-noise processing may involve related aspects of working memory, absence of significant association between FEST-S and speech performance may in part reflect colinearity among the metrics. Finally, no significant associations were found between FEST-S scores and either hearing sensitivity or age, despite the relatively broad age range of the older participants in the analyses.

## Experiment 2

This experiment investigated the effects of semantic context on the perception of environmental sounds in listeners with cochlear implants. To separate contributions of CI processing-related distortions from those of other user variables that can influence the involvement of semantic context, two groups were examined: YNH adults tested with CI vocoder simulations (CIV) and older experienced CI users. Prior to taking FEST-S, both groups practiced with FEST-I to achieve a similar level of group performance in identification of the individual sounds. Based on the findings of Experiment 1 in which YNH listeners outperformed MOI and MON listeners in terms of the beneficial effect of context, it was expected that the magnitude of the context effect would be greater for the YNH listeners in the CIV group than for the older adult CI users.

### Method

#### Ethics Statement

All methods were approved by the Institutional Review Board of the Rush University Medical Center, and all participants provided written informed consent.

#### Stimuli and Procedure

Experiment 2 closely followed the procedures of Experiment 1 except for the following modifications. Prior to testing listeners with sequences in FEST-S, both groups practiced with the individual environmental sounds in FEST-I. The practice consisted of initial FEST-I testing which was followed by three additional repetitions of FEST-I during which the 25 sounds were presented in random order with feedback. During these practice runs, if an individual sound was not identified correctly, the correct sound name appeared and the subject was required to listen to the sound three times before the next sound could be played. Following the three practice runs, during one final administration of FEST-I, subjects demonstrated moderately high identification accuracy with the individual environmental sounds: mean 75% correct (range 40–100%) for the CIV group, and mean 85% correct (range 69–100%) for the CI users.

Experiment 2 used BKB-SIN [69], instead of QuickSIN, for speech-in-noise testing of the CI users. As a test with simpler sentences in terms of their semantic context, BKB-SIN is more widely used with CI users. Neither SPIN-R, nor Reading Span were used in Experiment 2 due to a) lack of notable results with these tests in Experiment 1, and b), added time demands of Experiment 2 related the inclusion of the FEST-I familiarization. As in Experiment 1, participants in CI groups were administered MoCA and LNS. These tests however were not administered to participants in the CIV groups who tend to produce ceiling effects with a limited performance range on these tests. Except for three CI participants who completed the full protocol during a single visit, Experiment 2 was conducted during two separate sessions within a single week.

All FEST stimuli were presented to CIV subjects diotically via Sennheiser HD250 headphones in a sound-treated room at 75 dB SPL. Prior to presentation to CIV listeners, all FEST stimuli were modified with a vocoder to simulate effects of CI processing. Their spectral resolution was reduced to four frequency bands, using the spectral-degradation techniques of previous studies [50, 56, 58, 70]. The stimuli were 1) filtered with 6th order Butterworth filters (overlapping at - 3dB) into four log-spaced frequency bands within the 300–5500 Hz range; 2) envelopes from each band were obtained via half-wave rectification followed by lowpass filtering at 160 Hz; 3) white noise was modulated with the envelope of each band and filtered using the original filter settings; and 4) the four modulated noise bands were combined.

CI users were similarly tested in a sound-treated booth. However, all FEST and speech stimuli were presented to them unprocessed in a sound field at 70 dB SPL at the position of the listener head. Sound presentation was through a single loudspeaker positioned at 45 degrees to the implanted ear of each participant, who was sitting one meter away. The lower presentation level used with CI than CIV listeners was chosen to minimize potential input distortions that could have resulted from the application of automatic gain control of the CIs. Furthermore, the nonimplanted ear was occluded with an E-A-R Classic- AQ10 foam earplug (NRR 29 dB) to avoid potential residual hearing effects from the contralateral side.

#### Subjects

Two groups of listeners participated in Experiment 2. One group included 19 young adults (two males; age range: 20–26 yrs; mean 23 yrs) with normal audiometric thresholds (≤15 dB HL). The second group included eight postlingually deafened experienced CI users (three males; age range: 25–68; mean 54 yrs) with the average implanted-ear four-tone PTA (0.5, 1.0, 2.0, 4.0 kHz) equal to 28.1 dB (*SD* = 7.4 dB). Demographic characteristics of the CI users are listed in Table 7. For the CI users, mean implant experience was 3.6 years, with a minimum of 1 year of daily use. On average, the interval between identification of a severe-to-profound hearing loss to implantation for these subjects was 7.4 years. The average age of onset of a severe-to-profound hearing loss was 43.7 years (range: 16–54 yrs). All CI users had developed oral language skills prior to the onset of a severe-to-profound hearing loss and their primary mode of communication was oral.

**Table 7:** Cochlear implant listeners’ characteristics.

	Mean (SD)	Median	Range
**Age**	54.2 (12.8) yrs	56.5 yrs	25.0–68.0 yrs
**Age Severe-to-Profound Hearing Loss Identified**	43.2 (12.3) yrs	45.0 yrs	16.0–53.5 yrs
**Age at Implantation**	50.5 (13.6) yrs	52.0 yrs	21.0–66.0 yrs
**Duration of CI use**	3.6 (2.5) yrs	3.0 yrs	1.3–9.0 yrs
**PTA with CI**	27.71 dB HL (8.35)	27.5 dB HL	15–43.3 dB HL
**MoCA**	26.88 points (2.9)	27.5 points	21–30 points
**LNS**	10.63 points (2.77)	11 points	6–15 points
**BKB-SIN**	7.8 dB SNR 50 (4.85)	5.7 dB SNR 50	3–16 dB SNR 50

Characteristics of the cochlear implants (CI) users of Experiment 2, along with audiometric and cognitive test results.

### Results and Discussion

Following the analysis of ratings of sequence coherence, FEST-S scores obtained from CIV and CI participants were analyzed using the same three scoring metrics as in Experiment 1, with percent-correct scores submitted to an arcsine transform before data analysis. In addition, accuracy scores of CIV and CI participants, along with the three groups from Experiment 1, were evaluated to examine the effect of sound serial position within sequences on identification accuracy (i.e., primacy and recency effects). Next, response timing for the rating and identification tasks was examined to gain further insight into the effect of coherence in different subject groups. Lastly, a correlational analysis was conducted to evaluate possible associations between FEST-S and tests of working memory and cognition.

#### Coherence Ratings

Both CIV and CI subjects rated semantically coherent sequences as being more likely to occur at the same place or time than semantically incoherent ones (0.69 and 0.79 vs. 0.26 and 0.33, respectively). These ratings closely correspond with those obtained from the MON and MOI listeners in Experiment 1 (Table 2).

#### Context Effects: FEST-S

As can be seen in Fig 2, the performance of CIV and CI listeners on coherent and incoherent sequences was highly comparable. Three separate repeated-measures ANOVAs with group as a between-subject and context as a within-subject factor revealed a significant main effect of context with each of the three metrics (LC: *F* (1, 25) = 56.20, *p* < .001, η_p_^2^ = .69; OC: *F* (1, 25) = 24.26, *p* < .001, η_p_^2^ = .49; SC: *F* (1, 25) = 13.15, *p* = .001, η_p_^2^ = .35), but without significant differences between the groups (*p* = .18) or significant interaction between group and sequence type (*p* > .6). These results suggest that both CI and CIV listeners utilized the semantic information available in the sounds composing the test sequences to a similar degree. Being able to identify individual sounds with relatively high accuracy, both groups showed the capacity to benefit from semantic context despite differences in age, experience with CI-type processing of the sensory input, or history of hearing loss.

Additional analyses compared the performance of CI users with that of MON and MOI listeners, who, on average, provided a closer comparison in age than did the CIV group, and also performed similarly on the tests of cognitive abilities (i.e., MoCA and LNS). With one exception, CI users were middle-age to older adults, overlapping with the age range of the participants in the MON and MOI groups of Experiment 1. The MOI and MON subjects were combined as a single group due to lack of significant differences in their FEST-S performance, and were compared with CI users in six independent-samples t-tests (two, with either coherent and incoherent sequences, for each scoring metric—LC, OC, SC). Across tests, there were no significant effects (*p* > .45 for all comparisons), indicating comparable performance between CI listeners and listeners of a similar age range with either normal hearing or a mild-to-moderate hearing loss. This finding suggests that CI users were able to effectively utilize electrical stimulation from their implants to perform higher-order semantic information processing on auditory scenes and obtain a perceptual benefit from context, comparable to that of their MON and MOI peers.

Comparison between YNH and CIV listeners, two similar groups distinguished by the signal processing performed on the environmental sounds presented to the CIV group, indicated that sensory degradation introduced by cochlear implants can impede the processing of the semantic information in auditory scenes. Although both groups were able to benefit from semantic context in the coherent sound sequences, there was a general trend for YNH listeners to perform better than CIV listeners in all conditions. Six separate independent-samples t-tests (two sequence types by three scoring metrics) with a Bonferroni correction showed significant differences, *p* < .008 (0.05/6) in two scoring metrics. For the LC metric, the groups differed only in the coherent sequences, while for the SC metric the groups differed with both coherent and incoherent sequences. This indicates that the contextual benefit derived by CIV listeners was somewhat attenuated by signal distortion, when compared to their normal-hearing peers who listened to undistorted sequences. However, it may be that experienced CI listeners, who, unlike CIV listeners, had years of practice with distorted environmental sounds, could develop an adaptive strategy to maximize the perceptual benefits of semantic context in auditory scenes. The result of serial position analysis, described next, provides some support for this possibility.

#### Serial Position Effects

Identification of individual sounds in FEST-S sequences was further examined in terms of their serial position in the sequence. This analysis was performed for the OC metric only since it provided the largest performance range across groups and was deemed most informative. Differences in sound identification accuracy were expected to follow a typical U-shaped function which reflects superior recall of items occurring earlier and later in the sequence (i.e., primacy and recency effects) [71]. Of particular interest in this study was the relative magnitude of any such position order effect for coherent and incoherent sound sequences. A 2 (coherence) x 5 (group) x 5 (serial position) repeated-measures ANOVA revealed main effects of all three factors: coherence (F(1,67) = 83.7, *p <* .001, η_p_^2^ = .56), serial position F(4,268) = 42.87, *p* < .001, η_p_^2^ = .39, and group (F(1, 67) = 847, *p <* .001, η_p_^2^ = .93). A number of significant interactions were also observed: group and serial position (F(4,67) = 4.5, *p* < .001, η_p_^2^ = .21), coherence and position (F(1,67) = 11.87, *p <* .001, η_p_^2^ = .15, and a three-way interaction of coherence, position and group (F(4,67) = 2.07, *p <* .001, η_p_^2^ = .11). These interactions could result from the nonmonotonic pattern of sound identification accuracy across serial positions, and different performance on coherent and incoherent sequences by subjects in different groups. However, the coherence-by-group interaction was not significant (*p* = .53), possibly due to the lower accuracy of incoherent sequences in all five groups.

**Fig 3:**
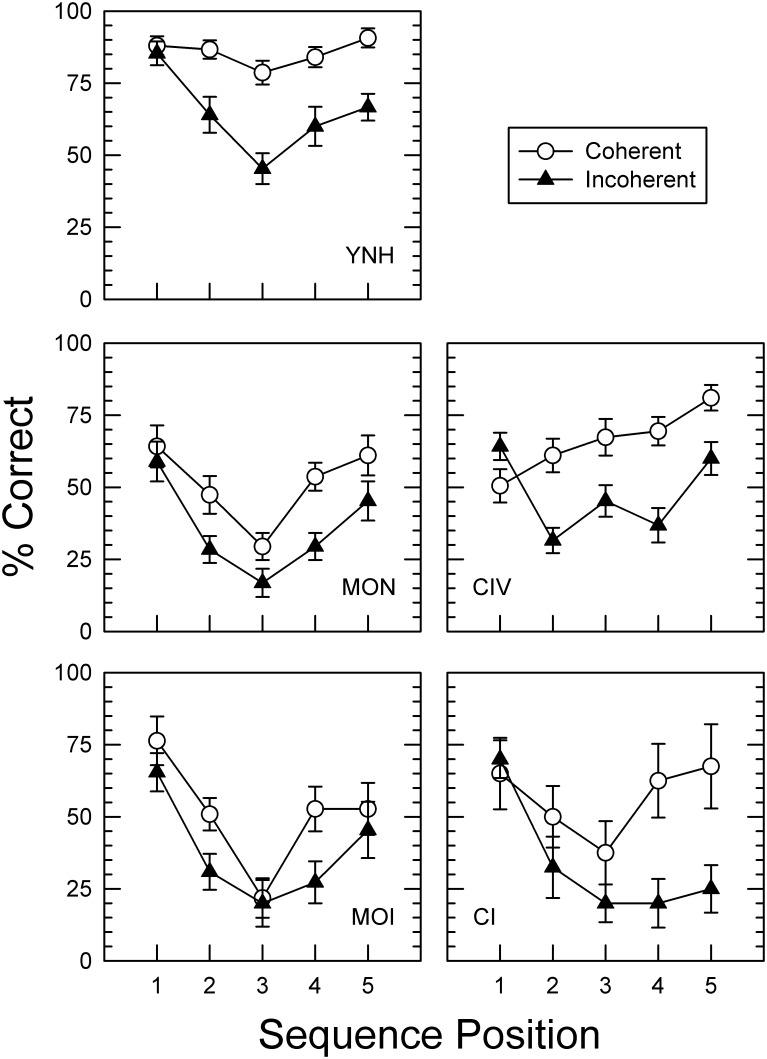
Serial position effects. Panels display performance accuracy of each group for all five serial positions of the individual environmental sounds comprising coherent and incoherent sequences. With some exceptions in CIV and CI groups, better performance for environmental sounds that occur early and late in the sequence can be seen with both coherent and incoherent sequences. Performance for coherent sequences is also generally better than incoherent sequences. Notably, CI users demonstrate the recency effect only for the coherent sequences, in which they could use contextual information, while the effect is absent for incoherent sequences. In all other groups, recency effects are evident for both coherent and incoherent sequences.

Interestingly, there were also clear differences in position order effects for coherent and incoherent sequences. Overall, sounds in incoherent sequences were recalled less accurately than coherent sequences. However, the differences in accuracy between coherent and incoherent sequences varied with sound position (Fig 3). Identification of the first sound was quite similar in both coherent and incoherent sequences, while identification of subsequent sounds in the sequences was lower for incoherent than for coherent sequences. With some exceptions in the MOI groups, this pattern of sound position differences was observed in all groups, indicating the positive effect of semantic context for identification of later coming sounds. Unexpectedly, in one group, CIV, identification accuracy of semantically coherent sequences did not follow the more typical U-shaped pattern, but was rising monotonically with sound position. Furthermore, in the CI group, the recency effect was absent for incoherent sound sequences, while it was present for coherent sequences.

It could be proposed that a simple measure of the difference in accuracy of identification of the last sound in coherent and incoherent sequences could indicate how much listeners may benefit from the preceding context. The greater the difference, the more semantic context provided by the previous sounds in the sequence has contributed. As can be seen in Fig 3, MON and MOI participants exhibited the least contribution of semantic context, while CI participants who had the greatest difference in the identification accuracy of the last sound in the sequence, derived the highest benefit from the preceding semantic context. It is also possible that rather than benefiting from context, CI users were more negatively affected by the lack of context in incoherent sequences. In either case, the reduced recency advantage for the last stimuli in the incoherent sequences may reflect the influence of the preceding context on the perceptual processing and memory access during categorization of environmental sounds. Overall, the interaction between sequence coherence with the effect of serial position indicates that the difference in identification accuracy for coherent and incoherent sequences increases over time as more semantically coherent sounds are presented for the former and not the later.

#### Response Timing on Rating and Identification Tasks

Additional analyses were conducted to evaluate the effect of coherence on the speed of performing ratings and identification tasks in each group. Generally, faster timing was observed in responses to coherent than incoherent sequences for both tasks (Table 8). Two separate 2 (coherence) x 5 (group) repeated-measures ANOVAs were performed on the logarithmically transformed time intervals required to complete first the rating task and then the identification task. Both ANOVAs revealed significant main effects of coherence (F(1,66) = 10.94, *p* < .002, η_p_^2^ = .14 for ratings, F(1,66) = 21.29, *p* < .001, η_p_^2^ = .24 for identification) and group (F(1, 66) = 1015.9, *p* < .001, η_p_^2^ = .94 for ratings, F(4, 66) = 91.6, *p* < .001, η_p_^2^ = .94 for identification), but no interactions. Overall, for both tasks, listeners took longer to rate incoherent than coherent sequences, with younger listeners in both YNH and CIV groups being significantly faster than older, hearing-impaired and CI listeners. The absolute differences in timing between younger and older listeners are quite striking as older listeners took nearly twice as long to complete both tasks, while also demonstrating inferior accuracy to YNH listeners. It is also worth noting that young adults in the CIV group listening to vocoded CI simulations were nearly as fast as their YNH peers listening to undistorted sounds. This suggests that the timing differences between groups were more influenced by participants’ age than the quality of sensory input.

**Table 8:** Average time in seconds taken to complete rating and identification tasks.

		Experiment 1			Experiment 2	
		YNH	MON	MOI	CIV	CI
**Rating**	**Coherent Sequences**	2.61 (.31)	5.48 (.73)	5.64 (.73)	2.79 (.29)	6.86 (.76)
	**Incoherent Sequences**	3.15 (.37)	5.6 (.52)	7.78 (.71)	3.09 (.33)	7.62 (1.16)
**Identification**	**Coherent Sequences**	35.39 (2.75)	85.81 (8.91)	74.99(6.71)	33.35 (2.38)	57.38 (11.38)
	**Incoherent Sequences**	41.49 (4.26)	87.77 (8.28)	84.0 (9.7)	40.8 (3.94)	77.22 (10.33)

Average time in seconds taken to complete rating and identification tasks for listener groups in both experiments: young normal-hearing (YNH), middle-to-older normal-hearing (MON), middle-to older hearing-impaired (MOI) listeners, with vocoder-simulated implants (CIV) and cochlear-implant users (CI). Standard errors are shown in parentheses below the average response time for each entry.

#### Correlations of FEST-S with Speech and Working Memory

Summary descriptions of scores on the tests of cognitive status, working memory, and speech-in-noise ability for CI users are listed in Table 7. The MoCA and LNS results for CI users were close to and not statistically different from those of the MON and MOI listeners of Experiment 1 (p = .57 for MoCA and p = .17 for LNS). Speech-in-babble thresholds from BKB-SIN testing of the CI users ranged from 3 to 16 dB, with an average threshold of 7.8 dB. This wide performance range obtained in speech-in-noise testing is consistent with past results of speech perception by CI users [72, 73].

Correlation analyses were conducted to examine if the use of semantic context in the perception of auditory scenes was associated with the speech-in-noise abilities of CI users. As in Experiment 1, only the OC metric was used to represent FEST-S performance. As shown in Table 9, BKB-SIN performance correlated strongly and significantly (*p* < .05) only with the scores for the coherent FEST-S sequences, while a moderate correlation with the incoherent sequences was not significant. This difference in correlation patterns for coherent and incoherent sequences suggests involvement of semantic context in the perception of both speech-in-noise and sounds in auditory scenes, potentially as part of a perceptual strategy to optimize performance. In contrast, correlations of working memory LNS scores to sequence identification approached significance (*p <* .*1*) only with the incoherent sequences, suggesting a greater role of working memory abilities in listening tasks when listeners cannot rely on contextual cues. Although the significance criterion of *p* < .1 is more liberal that what is typically reported, it was deemed appropriate owing to the exploratory nature and novely of this approach with CI users. Listener age did not significantly correlate with either FEST-S measure or BKB-SIN scores.

**Table 9:** Correlational analysis of cochlear implant listeners’ performance.

	Coherent Sequences	Incoherent Sequences
**Age**	-.17	-.47
**PTA**	-.25	.04
**BKB-SIN**	-.80**	-.64
**MoCA**	.53	.67*
**LNS**	.57	.70*

Pearson correlations between order-correct scores on coherent and incoherent sequences of cochlear implant (CI) listeners, with age and audiometric results.

** indicates significance at *p* < 0.05,

* indicates significance at *p* < 0.1.

## General Discussion

Successful navigation of real-world environments involves two interdependent perceptual skills: (1) identification of the objects and events in one’s vicinity, and (2) awareness of the relationships among these objects and events. For example, the sound of a barking dog may signal a coming visitor and could be preceded or followed by a doorbell. Honking may be followed by screeching tires, alerting listeners to the possibility of an accident. Running footsteps followed by a big splash may signal someone jumping into the water. These skills, ubiquitous in both visual and auditory modalities, aid people in constant monitoring of the environment, focusing of attention, and prediction of future events [3, 16, 31, 32, 37]. The present findings demonstrate that adult listeners of varying age and hearing abilities, including CI users, can utilize semantic context to improve the perception of environmental sounds in auditory scenes. However, the benefits of semantic context vary across listener groups as indicated by the three scoring metrics. When scoring only the least stringent aspects of the task, identification of correct sound labels regardless of the order in which they appear (i.e., the LC metric), all listener groups derived comparable benefit from the available semantic context. However, when the ability to reconstruct the order of sounds in a sequence was also assessed with the OC and SC metrics, YNH listeners outperformed older normal-hearing and older hearing-impaired listeners.

The better performance of YNH listeners may result from a combination of factors. In addition to better hearing sensitivity, YNH listener typically tend to demonstrate higher sensitivity to spectral and temporal variation, and greater cognitive capacity than older adults [41, 42, 47, 56]. Although working memory tests were not administered to the YNH listeners studied with either unprocessed or vocoded sound sequences, the current finding that auditory working memory, as measured by LNS, was a covariate in the perception of FEST-S sequences by MON, MOI and CI groups provides some support for this conjecture. Furthermore, as an independent predictor of performance, LNS working memory scores appeared to play a greater role for incoherent sound sequences when semantic context did not facilitate sound identification. On the other hand, the results of the Reading Span test, administered in the visual modality, were not predictive of environmental sound performance. This result might indicate differences in the processing of information in the visual vs. auditory modality, a decrement in the auditory acuity of older adults, or suggest dissociation between the semantic-linguistic processing of sentences and nonlinguistic environmental sounds.

Nevertheless, auditory working memory capacity as assessed by LNS, and a broader range of cognitive abilities, assessed by MoCA, may be involved in mediating the relationship between performance on FEST-S and speech perception in noise. Shared variance of these tests was a predictor of FEST-S scores for both coherent (Table 5) and incoherent (Table 6) sequences in Experiment 1. Moderate-to-high correlations between FEST-S and BKB-SIN scores for both coherent and incoherent sound sequences obtained in Experiment 2 with CI users may be further indicative of shared perceptual processes involved in the perception of auditory scenes and speech in noise. This finding is consistent with previous reports of the involvement of working memory in speech perception by older and hearing-impaired adults [38], and extends the previous results to the perception of environmental sounds in auditory scenes. On the other hand, unlike findings from speech perception research [5], the results do not indicate that older or hearing-impaired listeners rely on contextual information to a greater degree than YNH adults. This is especially underscored by the variation in the serial position effects across groups shown in Fig 3, where the differences between coherent and incoherent sequences for sounds that occurred at the end the sequence were greater for younger listeners and those with cochlear implants (YNH, CIV and CI) than older listeners with or without hearing loss (MON or MOI). Since final sounds would be most affected by the preceding semantic context, the greater identification accuracy of final sounds in coherent over incoherent sequences suggests greater reliance on contextual information. However, this discrepancy in context effects with speech findings may also reflect differences in speech and environmental sound perception which characterize different listener populations, result from the difference inherent in the assessment instruments used across studies, reflect the absence of masking noise in the present FEST protocol, or arise due to a generally younger age of the older listeners of the current participant sample [74]. It is, however, worth noting that the overall magnitude of the context effect in FEST-S as shown in Fig 2 is greater in YNH listeners than in other groups, being closely followed by CI listeners.

There are several additional factors that may have contributed to the strong facilitatory effects of semantic context found in the present study. The current sound identification procedure relied on word labels, and thus involved linguistic coding of stimuli names. Such explicit linguistic coding of the stimuli might have been enhanced since prior to identification, listeners rated the coherence of each stimulus sequence. Performing additional semantic operations on the stimuli during rating, which also further extended the time interval between stimulus presentation and identification of individual sounds, could conceivably increase the reliance on word labels for more efficient memory processing. Furthermore, the present results were obtained when all stimuli were presented in quiet. The addition of background noise, even without any identifiable semantic content, could also affect the strength of the context effect—a possibility consistent with prior research [13, 38].

The magnitude of the context effect might have also been affected by the order of individual sounds in the coherent sequences. Some sequences of individual sounds may be more statistically probable or semantically coherent than others. For example, snoring may be more likely to be heard before a ringing alarm clock than after. Although present results do not provide any indication about possible contributions of sound order, manipulations of sound position within sequences may elucidate the mechanisms behind the facilitatory context effects. It is possible that the context effect was facilitated by a temporally unfolding script-like template for coherent sequences in which each consecutive sound provides a certain degree of priming for the immediately following sounds. Alternatively, if the semantic context effect is based on the relatively long lasting activation of semantic categories corresponding to all of the individual sounds in a given sequence, the order of sounds may not be as important.

Overall, the current results are in agreement with prior environmental sound studies in showing that semantic context can have a facilitatory effect on environmental sound perception [11, 12]. Identification of specific sounds, and of their temporal position in the sequence, is improved when surrounding sounds form a semantic memory network, which can be activated by any of the member sounds [33, 37]. On the other hand, other work has shown that this effect may be level-dependent and that semantic context can also facilitate sound segregation when a specific sound is semantically incongruent with the rest of the auditory scene [13, 15, 38]. It appears that depending on task requirements (i.e., what the individual subject is listening for) semantic context can aid in either highlighting the sounds that do not go together or facilitating identification and memory encoding of the sounds that do go together. Such varied effects of semantic context align with previous theoretical considerations that form the tetrahedral model of perceptual experiments [75, 76]. In this model, the outcome on any perceptual task is viewed as the result of at least four major interacting factors: stimulus materials, listener characteristics, the nature of experimental tasks, and the specifics of the experimental context such as instructions and setting. Any one of these four factors may alter the experimental outcome. Thus, depending on the requirements of a specific listening task and the experimental setting, semantic context can be used to either draw attention to specific sounds that are different from other immediate sounds or to improve identification of individual sounds that form a semantically coherent auditory scene.

## Conclusions and Future Directions

Overall, based on the present findings, a brief environmental sound test, FEST, appears to be effective in detecting semantic context effects in listeners of varying age and hearing abilities. The three scoring metrics which differentially assess response accuracy provide further flexibility in scoring, and can be useful when examining semantic context effects in diverse listener populations. In the current study, all listener groups demonstrated robust use of semantic context in the perception of environmental sound sequences. At the least stringent level of assessment, LC, which did not take into account correct placement of sound in the sequence, the ability to identify sounds in short auditory scene-like sequences was not affected by any of the potentially detrimental factors: age, presbycusis or listening through a cochlear implant. Participants were able to utilize the tacit knowledge of probabilistic relationships among different environmental sounds forming the semantically coherent sequences. The use of semantic context thus provides an important advantage in the perception of environmental sounds. Furthermore, auditory working memory, along with other cognitive abilities, appears to play a role in maximizing performance in the perception of environmental sounds in auditory scenes.

The ability to utilize semantic context in auditory scenes may, however, be reduced in other listener populations. For instance, prelingual CI users who have not developed typical auditory cognitive capacity in childhood or individuals with certain central-processing disorders or cognitive impairments may have difficulty integrating information across the semantic categories associated with specific environmental objects and events. Finally, as a short instrument for the assessment of higher-order auditory cognitive abilities that rely on environmental sounds, FEST can be potential useful in cognitive and auditory assessments of populations with limited command of the English language. To that end, present efforts are directed toward the development of a version of FEST that uses pictures rather than word labels for indicating subject responses, as well as a version with a variable number of environmental sounds in the sequences to accommodate gradual perceptual learning. This gradation in terms of working-memory load and semantic difficulty can increase its utility in the assessment of the auditory cognition of children and adults with limited literacy. Other applications may include aural rehabilitation programs to improve real-world listening skills in CI users and older adults, either with or without a hearing loss.
